# A novel peptide (GX1) homing to gastric cancer vasculature inhibits angiogenesis and cooperates with TNF alpha in anti-tumor therapy

**DOI:** 10.1186/1471-2121-10-63

**Published:** 2009-09-09

**Authors:** Bei Chen, Shanshan Cao, Yingqi Zhang, Xin Wang, Jie Liu, Xiaoli Hui, Yi Wan, Wenqi Du, Li Wang, Kaichun Wu, Daiming Fan

**Affiliations:** 1 State Key Laboratory of Cancer Biology and Xijing Hospital of Digestive Diseases, the Fourth Military Medical University, Xi'an, Shaanxi, PR China; 2 State Key Laboratory of Cancer Biology and Biotechnology Centre, the Fourth Military Medical University, Xi'an, Shaanxi, PR China

## Abstract

**Background:**

The discovery of the importance of angiogenesis in tumor growth has emphasized the need to find specific vascular targets for tumor-targeted therapies. Previously, using phage display technology, we identified the peptide GX1 as having the ability to target the gastric cancer vasculature. The present study investigated the bioactivities of GX1, as well as its potential ability to cooperate with recombinant mutant human tumor necrosis factor alpha (rmhTNFα), in gastric cancer therapy.

**Results:**

Tetrazolium salt (MTT) assay showed that GX1 could inhibit cell proliferation of both human umbilical vein endothelial cells (HUVEC) (44%) and HUVEC with tumor endothelium characteristics, generated by culturing in tumor-conditioned medium (co-HUVEC) (62%). Flow-cytometry (FCM) and western blot assays showed that GX1 increased the rate of apoptosis from 11% to 31% (*p *< 0.01) by up-regulating caspase 3 expression level. A chorioallantoic membrane assay indicated that GX1 could suppress neovascularization *in vivo*, with the microvessel count decreasing from 21 to 11 (*p *< 0.05). When GX1 was fused to rmhTNFα, GX1-rmhTNFα selectively concentrated in the gastric cancer vasculature, as shown by enzyme-linked immunosorbent assay, immunofluorescence and emission-computed tomography. *In vitro *MTT and FCM assays showed that, compared to rmhTNFα alone, GX1-rmhTNFα was more effective at suppressing co-HUVEC proliferation (45% vs. 61%, *p *< 0.05) and inducing apoptosis (11% vs. 23%, *p *< 0.05). In a tumor formation test, GX1-rmhTNFα more effectively inhibited tumor growth than rmhTNFα (tumor volume: 271 mm^3 ^vs. 134 mm^3^, *p *< 0.05), with less systemic toxicity as measured by body weight (20.57 g vs. 19.30 g, *p *< 0.05). These therapeutic effects may be mediated by selectively enhanced tumor vascular permeability, as indicated by Evan's blue assay.

**Conclusion:**

GX1 had both homing activity and the ability to inhibit vascular endothelial cell proliferation *in vitro *and neovascularization *in vivo*. Furthermore, when GX1 was conjugated to rmhTNFα, the fusion protein was selectively delivered to targeted tumor sites, significantly improving the anti-tumor activity of rmhTNFα and decreasing systemic toxicity. These results demonstrate the potential of GX1 as a homing peptide in vascular targeted therapy for gastric cancer.

## Background

Ever since the essential role of angiogenesis in tumor formation and metastasis was proposed by Folkman in 1971, increasing attention has been paid to vascular targeted therapy [1-3]. The vasculature is an attractive target because vascular endothelial cells are more genetically stable than tumor parenchymal cells and less likely to acquire drug resistance, and vascular targets on endothelial cells are readily accessible to systemically delivered agents [4-6]. Based on these advantages, efforts have focused on identifying specific molecules expressed on the surface of tumor vascular endothelial and perivascular cells [7,8]. Finding such tumor vascular targets may help make anticancer drugs more selective, through their targeted delivery, thus providing higher therapeutic efficiency while simultaneously decreasing systemic toxicity.

With this goal, we previously used *in vivo *screening of a phage-displayed peptide library to identify a cyclic 7-mer peptide, CGNSNPKSC, called GX1, which binds specifically to the human gastric cancer vasculature [9]. Immunohistochemical staining, enzyme-linked immunosorbent assay (ELISA), and immunofluorescence confirmed the targeting activity of GX1 peptide, indicating that GX1 might be used as a novel vascular marker for human gastric cancer [10]. The potential bioactivities that might accompany the targeting function of GX1, and how it might be combined with other agents for antitumor therapy, are investigated here. We conducted a series of tests to determine the effects of GX1 on vascular endothelial cells, and on tumor angiogenesis and growth. In addition, we fused GX1 to recombinant mutant human tumor necrosis factor (rmhTNFα), a variant of the TNFα cytokine that is well known for its potent antitumor activity and is less toxic than TNFα [11,12], to see if the fusion protein could achieve synergistic therapeutic efficacy. These studies provide important preclinical evidence for the use of GX1 in targeted antitumor therapy.

## Results

### GX1 inhibits endothelial cell proliferation *in vitro *by inducing apoptosis

**Figure 1:**
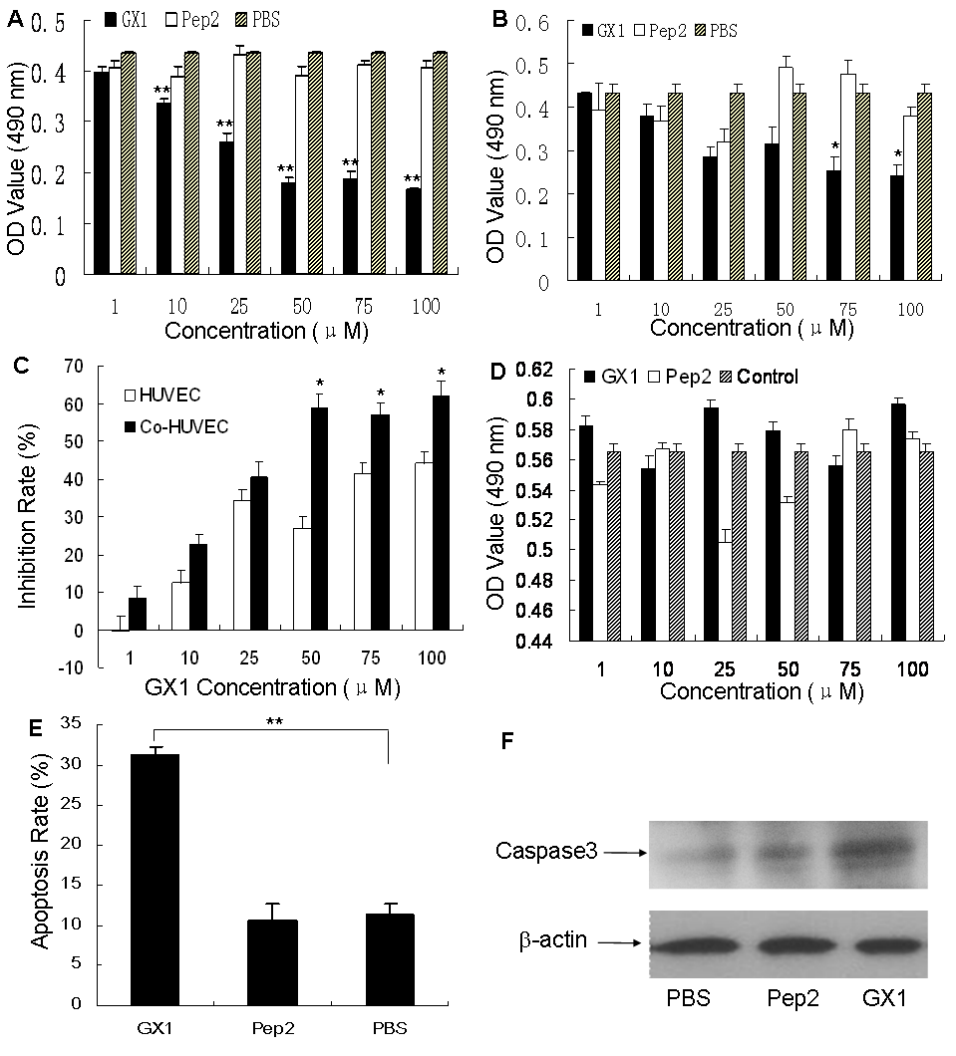
**GX1 inhibits cell proliferation of both co-HUVEC and HUVEC through induction of apoptosis**. GX1 was shown by MTT assay to suppress the proliferation of co-HUVEC (A) and HUVEC (B) in a dose-dependent manner. (C) GX1 had a greater inhibitory effect on co-HUVEC than HUVEC. (D) GX1 had no obvious effect on gastric adenocarcinoma SGC7901 cells. (E) GX1 at 50 *μ*M significantly enhanced apoptosis of co-HUVEC over PBS treatment. The results are reported as mean ± SD of three independent experiments. *Bars, SD*. * *p *< 0.05, ** *p *< 0.01. (F) GX1 at 50 *μ*M remarkably up-regulated caspase 3 expression level.

GX1 was tested for its ability to inhibit both endothelial cells and tumor cell proliferation by the tetrazolium salt (MTT) assay. The results showed that GX1 could reproducibly suppress, in dose-dependent manner, the proliferation of human umbilical vein endothelial cells (HUVEC) and HUVEC with tumor endothelial cell characteristics (co-HUVEC), generated by culturing the cells in tumor-conditioned medium [13]. Differences in the relative cell number between cells treated with GX1 and the control peptide (Pep2) were significant at concentrations of 10, 25, 50, 75 and 100 *μ*M for co-HUVEC (*p *< 0.01) (Figure 1A), and were significant at 75 and 100 *μ*M for HUVEC (*p *< 0.05) (Figure 1B). In addition, GX1-induced inhibitory effects were more obvious in co-HUVEC than in the non-tumor conditioned HUVEC (Figure 1C). In contrast, no such differences were detectable in gastric adenocarcinoma SGC7901 cells (Figure 1D), further demonstrating the selectivity of GX1.

Subsequently using flow-cytometry (FCM), it was found that GX1 (50 *μ*M), but not Pep2 (50 *μ*M), induced apoptosis in co-HUVEC compared to the phosphate-buffer saline (PBS) control (apoptosis rate: 31.2% vs. 11.4%, *p *< 0.01) (Figure 1E). However, assessment of cell cycle distribution by FCM showed no significant difference between the test and control groups. These results indicated that inhibition of vascular endothelial cell proliferation by GX1 was, at least in part, through induction of apoptosis. We further detected the expression level of the apoptosis related molecule caspase 3 by western blot. As shown in Figure 1F, the expression of cleaved caspase 3 was up-regulated in GX1 (50 *μ*M) treated co-HUVEC. In contrast, no changes were detected in Pep2 (50 *μ*M) or PBS treated cells. In brief, GX1 appeared to induce apoptosis of co-HUVEC by up-regulating the expression of caspase 3.

**Figure 2:**
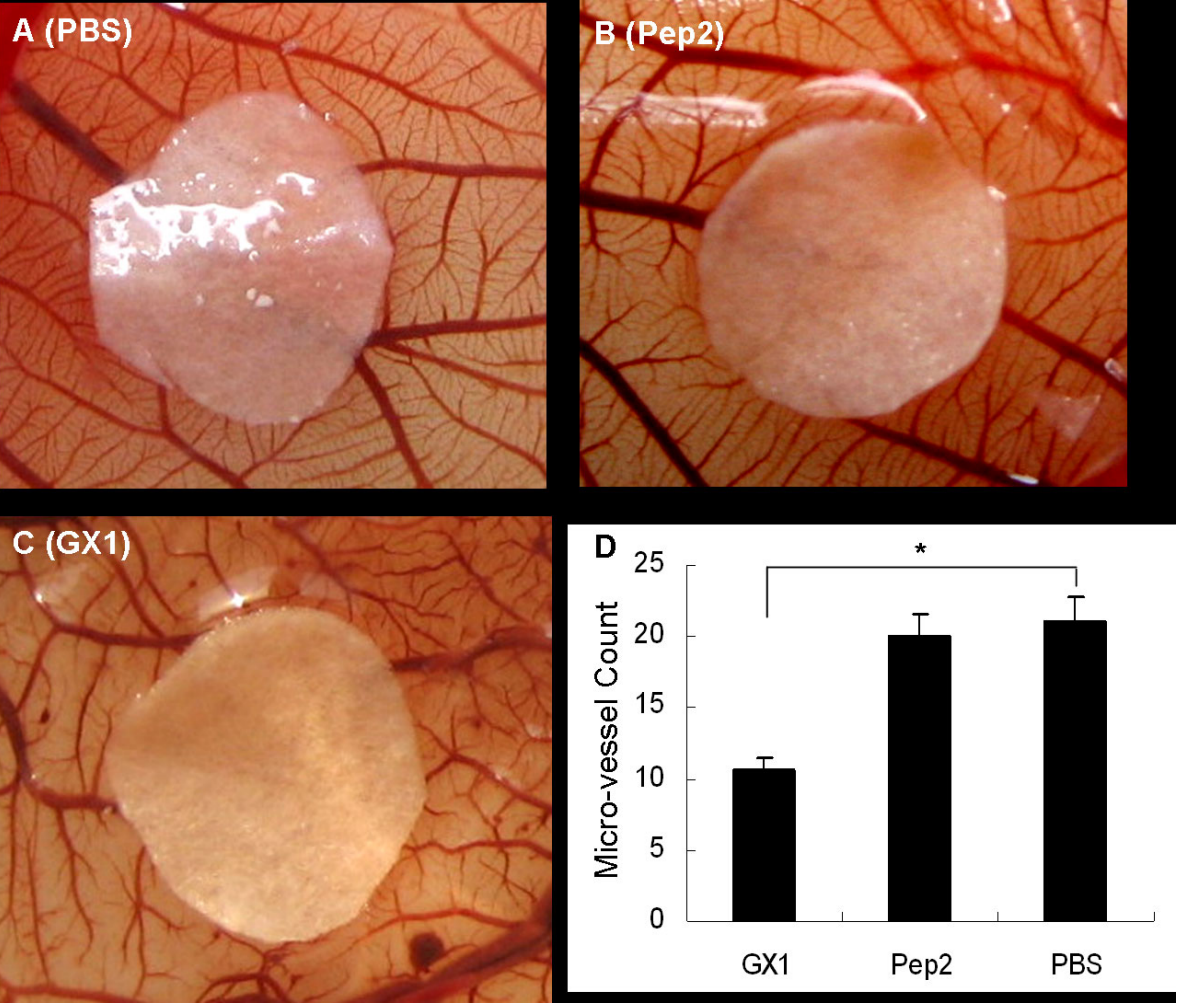
**GX1 inhibits angiogenesis in CAM assay**. (A-C) GX1 at 20 *μ*g (50 *μ*M) hampered neovascularization of fertilized eggs. Attenuated and tortuous microvessels are shown in the CAM, with fewer angiogenic vessels contacting the filter disks. (D) Number of microvessels contacting the disks. *Bars, SD*. * *p *< 0.05.

### GX1 inhibits angiogenesis *in vivo *by a chorioallantoic membrane (CAM) assay

Since GX1 could repress vascular endothelial cell proliferation *in vitro*, we carried out CAM assays to see if the peptide could inhibit angiogenesis *in vivo*. Disruption of angiogenesis was observed in GX1-treated chicken embryos, with attenuated and tortuous microvessels in the CAM and fewer angiogenic vessels contacting the disk, when compared to the PBS control group. No significant differences existed between the Pep 2 and PBS control groups, with both showing well-developed and leaf vein-like vascular nets (Figure 2).

### GX1 conjugated to rmhTNFα concentrates in gastric cancer

**Figure 3:**
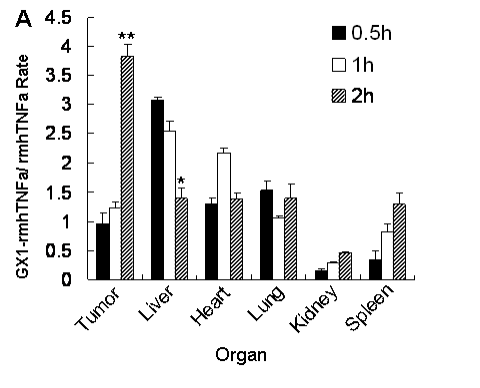
**Distribution of GX1-rmhTNFα in various organs**. GX1-rmhTNFα accumulated in tumor tissues over time and was gradually cleared from the liver. GX1-rmhTNFα/rmhTNFα rate = radioactivity of GX1-rmhTNFα relative to rmhTNFα. *Bars, SD*. *p < 0.05, **p < 0.01.

In addition to proapoptotic and anti-angiogenic activity, GX1 was also assessed for its ability to act as a targeting delivery vector in combination therapy for gastric adenocarcinoma. GX1 was fused to rmhTNFα as previously reported [14] and its tumor-targeted distribution was investigated. ELISA was used to analyze the amount of GX1-rmhTNFα in tumor and non-tumor tissues, and the results showed that GX1-rmhTNFα accumulated in tumor tissues over time. The radioactivity of GX1-rmhTNFα relative to rmhTNFα (GX1-rmhTNFα/rmhTNFα rate) significantly increased in tumor tissue from 0.95 at 0.5 h to 3.84 at 2 h (*p *< 0.01). In contrast, this ratio decreased in liver within 2 h after administration of the agent (*p *< 0.05), and no evident trend was observed in other major organs (Figure 3).

**Figure 4:**
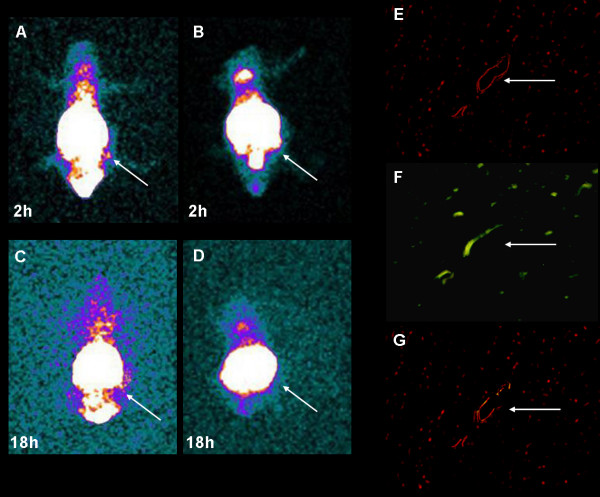
**GX1-rmhTNFα selectively concentrates at gastric cancer cells**. ^99^Tc^m^-GX1-rmhTNFα remained in tumor tissue from 2 h to 18 h after injection (A, C), while no such accumulation was seen in the ^99^Tc^m^-rmhTNFα group (B, D). Tumors are indicated by arrows. (E) Positive staining of CD31 in tumor vasculature (200×). (F) FITC-labeled GX1-rmhTNFα bound specifically to tumor vasculature (200×). (G) GX1-rmhTNFα colocalized with CD31 in tumor vasculature (200×). Arrow indicates a positively stained microvessel.

We took planar scintigrams of tumor-bearing mice injected with ^99^Tc^m^-GX1-rmhTNFα, to further confirm the *in vivo *tumor-targeting. Compared to ^99^Tc^m^-rmhTNFα (Figure 4B, D), the radioactivity in tumor tissue was much higher in the ^99^Tc^m^-GX1-rmhTNFα group (Figure 4A, C). The tumor/muscle radioactivity ratios of ^99^Tc^m^-GX1-rmhTNFα and ^99^Tc^m^-rmhTNFα were 8.24 and 1.42, respectively, at 18 h (*p *< 0.05). Simultaneously, immunofluorescence was used to detect the specific location of GX1-rmhTNFα. GX1-rmhTNFα, but not rmhTNFα, was found to co-localize with CD31 which was used as a positive control in tumor vasculature (Figure 4E-G). Collectively, these results suggest that the fusion protein acquired the ability to target tumor vessels and may lead to more selective drug delivery to tumors.

### GX1-rmhTNFα inhibits co-HUVEC proliferation *in vitro *by inducing apoptosis

**Figure 5:**
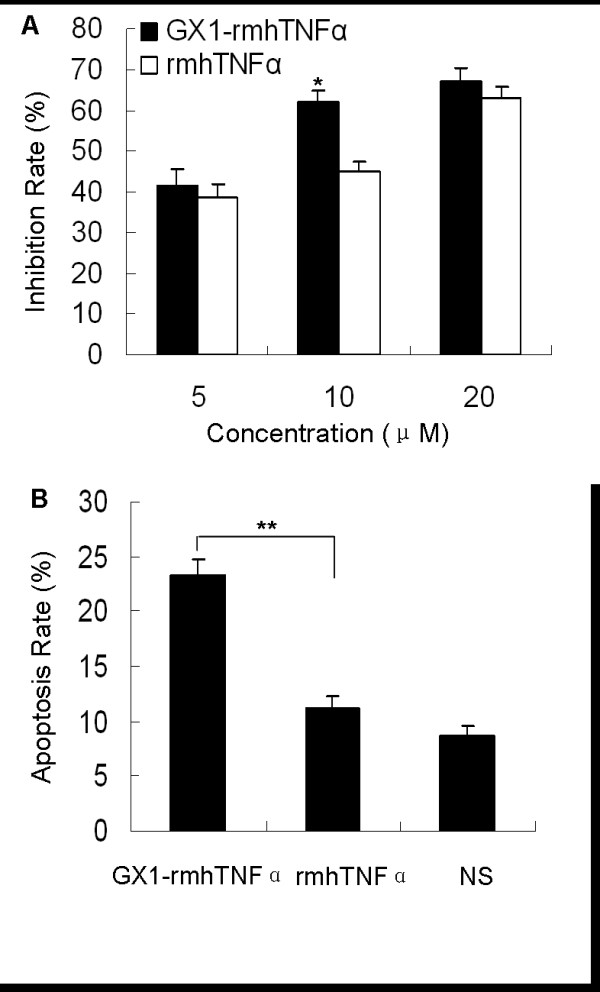
**GX1-rmhTNFα inhibits co-HUVEC proliferation through induction of apoptosis**. (A) GX1-rmhTNFα at 10 *μ*M more strongly inhibited co-HUVEC proliferation than rmhTNFα. (B) GX1-rmhTNFα at 10 *μ*M more strongly enhanced apoptosis of co-HUVEC than rmhTNFα. The results are reported as mean ± SD of three independent experiments. *Bars, SD*. * *p *< 0.05, **p < 0.01.

The MTT assay was used to detect the effects of GX1-rmhTNFα on endothelial cell proliferation, and showed that the fusion protein was significantly better at inhibiting co-HUVEC proliferation than rmhTNFα, with inhibition rates of 61% vs. 45% at 10 *μ*M (*p *< 0.05) (Figure 5A).

To address whether the decrease in cell number was due to apoptosis induced by GX1-rmhTNFα, FCM was used to determine the apoptosis rate in co-HUVEC. The results showed that the apoptosis rates induced by 10 *μ*M GX1-rmhTNFα or rmhTNFα were 23.4% and 11.2% respectively (*p *< 0.01) (Figure 5B). In contrast, no significant differences in cell cycle distribution were detected between the test and control groups. These results indicated that inhibition of vascular endothelial cells by GX1-rmhTNFα might be partly caused by induction of apoptosis.

### Effects of GX1-rmhTNFα on tumor growth *in vivo*

**Figure 6:**
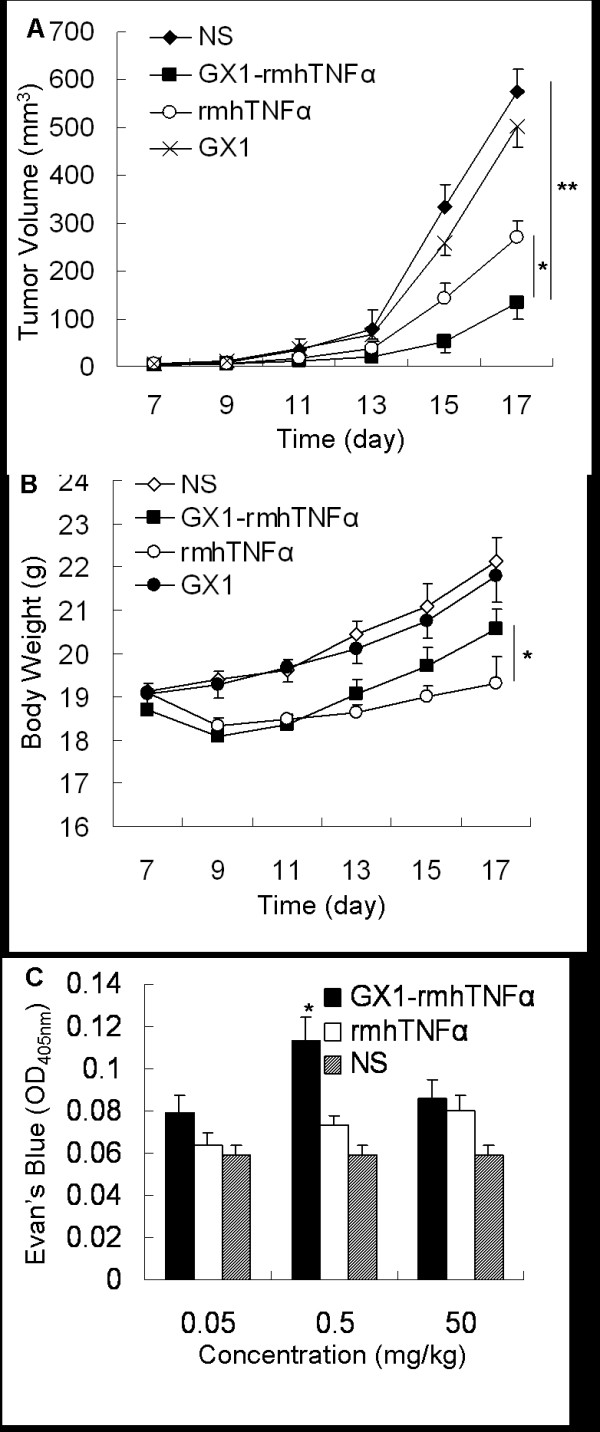
**GX1-rmhTNFα inhibits tumor growth with decreased systemic toxicity by enhancing tumor vascular permeability**. (A) GX1-rmhTNFα significantly delayed tumor growth. (B) GX1-rmhTNFα led to less weight loss than rmhTNFα. (C) GX1-rmhTNFα induced a greater leakage of the dye in the tumor parenchyma than rmhTNFα. *Bars, SD*. * *p *< 0.05, **p < 0.01.

Subsequently, using an *in vivo *tumor formation test, GX1-rmhTNFα was assayed for its effects on tumor growth. Nude mice bearing human gastric adenocarcinoma xenografts were injected intravenously on alternate days with GX1-rmhTNFα (0.5 mg/kg), rmhTNFα (0.5 mg/kg), GX1 (0.25 mg/kg to account for its lower molecular weight) or normal saline (NS). Mouse body weight, which is used as a major indicator of TNF toxicity [11,15], and tumor mass volumes were assessed over time. Tumor growth was significantly delayed by GX1-rmhTNFα treatment, and the average tumor volume of the GX1-rmhTNFα group was much smaller than that of the rmhTNFα group (134.33 mm^3 ^vs. 271.50 mm^3^, *p *< 0.05) (Figure 6A). In addition, at the end of the test, mice treated with GX1-rmhTNFα had a higher average body weight than those treated with rmhTNFα (20.57 g vs. 19.30 g, *p *< 0.05) (Figure 6B), suggesting that the fusion protein had less systemic toxicity than rmhTNFα alone.

Since TNFα is known to alter vascular barrier function, we performed Evan's blue assay to assess the effect of GX1-rmhTNFα on tumor perfusion. Compared to rmhTNFα-treated mice, the GX1-rmhTNFα treated group showed a greater leakage of Evan's blue dye in the tumor parenchyma. Differences were statistically significant at 0.5 mg/kg (0.113 vs. 0.073, *p *< 0.05) (Figure 6C). We therefore hypothesize that the GX1-rmhTNFα fusion protein selectively increases tumor vascular permeability and leads to higher local drug levels, which may play an important part in the antitumor mechanism of GX1-rmhTNFα.

## Discussion

To improve therapeutic indices and decrease systemic toxicity, more specific and selective anticancer agents that can discriminate between tumor and nonmalignant cells are urgently needed, along with the development of antitumor radiochemotherapy [16-18]. The discovery that angiogenesis plays a crucial role in tumor formation, and that vascular targeting approaches exhibit the advantages of easy accessibility and lower incidence of drug resistance, provides a possible path to creating these new anticancer agents [19,20]. Several studies have pursued this strategy, including the application of phage display technology to pan for peptides that bind specifically to defined tissue targets [8,21]. Using this technique, several homing peptides have been identified, including RGD, NGR and F3, and many have showed promising results for imaging diagnosis and treatment of various tumors in preclinical or clinical investigations [22-25]. Furthermore, some of these peptides have been conjugated to bioactive agents, including drugs, cytokines, procoagulant factors, photosensitizers and radionuclides, and have been included in antineoplastic therapies. Initial results of these studies showed more selective and targeted drug delivery and fewer side effects [7,21,26,27]. However, to date, no such peptide has been identified that targets human gastric cancer.

Previously, we used *in vivo *screening of a phage displayed peptide library to identify GX1, a cyclic 7-mer peptide CGNSNPKSC that binds specifically to the human gastric cancer vasculature [9]. Autoradiography on different cell lines confirmed the targeting activity of GX1 toward the gastric cancer vascular endothelium, by showing that the binding affinity of GX1 was significantly higher in HUVEC cultured in tumor-conditioned medium than in HUVEC cultured in non-conditioned medium. No specific binding was observed in the human gastric carcinoma cell line SGC7901 or in the immortalized gastric epithelial cell line GES cells [28]. Furthermore, immunohistochemical staining and immunofluorescence showed positive staining for GX1 in the vascular endothelium of human gastric adenocarcinoma, but not in heart, liver, muscle, spleen or normal gastric tissues [10,28]. In another study, using single photon emission computed tomography (SPECT), GX1 labelled with ^99^Tc^m^O_4_^- ^(^99^Tc^m ^-GX1) was observed to concentrate in tumor xenografts in nude mice [28]. Collectively, these results indicate that GX1 is a novel vascular marker of human gastric cancer, and may lead to a new way of imaging diagnosis and targeted gastric cancer therapy.

Since GX1 selectively targeted the vascular endothelium of gastric cancer, we investigated whether it had specific effects on tumor angiogenesis and growth. In this study, in addition to targeting, GX1 showed bioactivity by both MTT and CAM assay, inhibiting vascular endothelial cell proliferation and hampering neovascularization. To probe into the possible mechanisms of these effects, the cell cycle distribution, cell apoptosis and the expression level of apoptosis related molecule caspase3 were detected by FCM and western blot assays. Inhibition of vascular endothelial cell proliferation by GX1 was observed, at least in part, to be through the up-regulation of caspase 3 expression and the induction of apoptosis. Further tests including RT-PCR and gene microarray are underway to investigate the precise mechanisms.

*In vitro *analysis showed that HUVEC cultured in tumor-conditioned media partially acquire the characteristics of tumor vascular endothelial cells, such as enhanced tubule formation, cell proliferation, and migration [13,29]. Furthermore, some proteins like vascular endothelial growth factor receptor and the integrin αvβ3 may be up-regulated in co-HUVEC, as is the case for other cancer endothelia [6,30]. These findings lead us to the hypothesis that GX1 receptors are up-regulated in co-HUVEC, reflecting the case in tumor vessels, and that more receptors may lead to greater selective affinity and stronger anticancer effects. This hypothesis is consistent with the MTT assay results, in which GX1 showed more significant inhibitory effects on co-HUVEC than on the parental HUVEC culture that was not exposed to tumor-conditioned medium.

To assess the possibility of using GX1 as a targeted delivery vector in combination with another antitumor molecule for treatment of gastric cancer, GX1 was conjugated to rmhTNFα. TNFα is a well-known, antitumor cytokine whose clinical application is hampered by severe systemic toxicity [12,15]. The novel mutant cytokine rmhTNFα shows higher antitumor efficacy and has been approved for clinical use in China [12]. Our data showed that after fusion to GX1, rmhTNFα was selectively delivered to target tumor vasculature sites. Most important, GX1-rmhTNFα delayed tumor growth *in vivo*, with less loss of body weight compared to rmhTNFα alone (Figure 6A, B). These results indicated that more targeted and efficient antitumor activity might be achieved by combining GX1 with other anti-tumor agents (e.g. rmhTNFα), for a significant reduction in systemic toxicity.

Despite the encouraging results, some questions are still open, such as what the receptor is for GX1 on vascular endothelial cells, and how ligand-receptor interaction interferes with tumor angiogenesis. Further studies are underway to answer these questions, and several candidate receptor molecules have been obtained. Identification of the GX1 receptor will be a great help in understanding the mechanism of GX1 and will accelerate the development of clinical applications for GX1 in diagnosis and targeted treatment of gastric cancer.

## Conclusion

In conclusion, the data presented here, taken together with previously published results, demonstrate that GX1 is a novel vascular marker of human gastric cancer. For the first time GX1 is shown to have properties other than homing, including proapoptotic effects on vascular endothelial cells, and *in vivo *inhibition of neovascularization. Furthermore, when conjugated to rmhTNFα, GX1 selectively delivered the fusion protein to target tumor sites, leading to higher antitumor efficiency with less systemic toxicity. These findings demonstrate great potential for developing GX1 both as a targeted vector and as an antiangiogenic agent in the diagnosis and treatment of human gastric cancer.

## Methods

### Reagents, antibodies and peptides

Growth factors and Evan's blue were purchased from Sigma (St. Louis, USA). M200 basal culture media and low serum growth supplement (LSGS) were from Cascade Biologics (USA). Anti-CD31 polyclonal antibody was from ABcam (USA) and anti-TNF monoclonal antibody was from Sigma Aldrich (Saint Louis, USA).

GX1 peptide (CGNSNPKSC) was synthesized by GL Biochem (Shanghai) Ltd. A control peptide (Pep2) was created by randomly scrambling the amino acid sequence of GX1 while maintaining the disulfide bond to preserve the U-type structure (CNKSPSGNC). rmhTNFα was created by standard recombinant DNA techniques [11,31,32]. The GX1-rmhTNFα fusion protein was prepared as previously described [14].

### Cell cultures

HUVEC (Cascade Biologics, USA) and the human gastric cancer cell line SGC7901 were cultured as described [28]. Tumor conditioned medium (TCM) was prepared by incubating SGC7901 cells in M200 (free of LSGS) (~1 × 10^6^/ml) for 24 h. The medium was then removed, centrifuged (2000 × g, 10 min), filtered with a 0.22-*μ*m filter and diluted five times with M200 supplemented with LSGS. Tumor endothelial cells were generated by incubating HUVEC in TCM [29]. All cells were cultured at 37°C in a humidified chamber with 5% CO_2_.

### MTT, FCM and western blot assays

Proliferation of HUVEC and SGC7901 cells treated with various concentrations of tested agents was determined by MTT assay as described [33].

Apoptosis of co-HUVEC was detected by FCM analysis as described [34]. Cells were treated with GX1 (50 *μ*M) or GX1-rmhTNFα (10 *μ*M) for 48 h. Pep2 (50 *μ*M), PBS or rmhTNFα (10 *μ*M) were used as controls.

The expression level of caspase 3 was measured by western blot assay. The cultured cells were lyzed in modified RIPA buffer (0.05 M Tris-HCl, pH 7.4, 1% NP-40, 0.25% Na-deoxycholate, 0.15 M NaCl, 0.001 M Na_3_VO_4_, 0.001 M EDTA and 0.5% of protease inhibitor cocktail). The lysate was centrifuged at 10,000 × g, 4°C for 10 minute, and the supernatant was collected. Protein concentration was determined by the BCA protein assay (Pierce, Rockford, IL, USA). Proteins were separated by 10% SDS-PAGE and were transferred to PVDF membrane. western blot analysis was carried out using the following primary antibodies: anti-cleavage caspase 3 antibody (1:500; Abnova Corporation, Taipei, Taiwan) and anti-β-actin antibody (1:1000; Santa Cruz Biotechnology, Santa Cruz, CA, USA), followed by incubation with horseradish peroxidase (HRP) conjugated secondary antibody (Santa Cruz Biotechnology, Santa Cruz, CA, USA). The blots were visualized using enhanced chemiluminescence (ECL) kit (Amersham Pharmacia Biotech, Arlington Heights, IL, USA) according to manufacturer's instructions.

### Chorioallantoic Membrane (CAM) assay

Fertilized White Leghorn chicken embryos were randomly divided into three groups with seven embryos per group, and collected on day 3 into sterile containers for subsequent incubation at 37°C, 5% CO_2 _for 6 days. On day 9, sterilized Whatman filter discs impregnated with 10 *μ*l (20 *μ*g) GX1 were placed on the CAM. Pep2 (20 *μ*g) and PBS were used as controls. On day 11, the CAM was cut, fixed by acetone and viewed under a microscope. Neovascularization around the disk was quantitated by determining the number of angiogenic vessels within the CAM around the disk.

### *In vivo *distribution of GX1- rmhTNFα by emission computed tomography (ECT)

*In vivo *distribution of GX1-rmhTNFα in different organs of tumor-bearing nude mice was detected by enzyme-linked immunosorbent assay (ELISA, see below) and ECT. A suspension of SGC7901 cells was prepared at 1 × 10^7 ^cells/ml. A total of 0.2 ml of the cell suspension was implanted subcutaneously in the right upper flank of 4-6 week-old male nude BALB/c mice (animal centre of FMMU, Xi'an, Shannxi, China). Three weeks after injection, the mice were randomly divided into three groups with six mice per group, and treated with 0.25 mg/kg of GX1-rmhTNFα or rmhTNFα, or NS through the tail vein. The agents were allowed to circulate for 0.5 h, 1 h and 2 h before blood samples were taken from the eye, and tumors and major organs were removed. Tissue samples were homogenized and sonicated at 4°C, followed by centrifugation for 10 min at 12,000 × g. The supernatant was subjected to ELISA.

Simultaneously, GX1-rmhTNFα labeled with ^99^Tc^m^O_4 _^- ^was used for dynamic imaging in biodistribution studies[28]. Anesthetized animals were injected intravenously with 200 *μ*l ^99^Tc^m^-GX1-rmhTNFα or ^99^Tc^m^-rmhTNFα at 470-540 *μ*Ci per mouse. Planar and single-photon emission tomography images with a low-energy collimator were obtained, with 200,000 counts acquired per image at the indicated timepoints. Time-dependent biodistribution studies were carried out by sacrificing mice at 2, 8, and 18 h after injection. Tissue samples were removed at the end of the test. The radioactivity was determined with a gamma counter and decay-corrected to the time of injection. Results were calculated as injected dose (ID) per gram of wet tissue weight (ID/g tissue), converted to percent. GX1-rmhTNFα/rmhTNFα radioactivity rates of various tissues were determined from the corresponding ID/g tissue values.

### ELISA, immunohistochemical staining and immunofluorescence staining

The amount of GX1-rmhTNFα and rmhTNFα in tumor and other organs was quantified by ELISA kit (Department of Immunology, FMMU, Xi'an, Shannxi, China). The optical density at 470 nm was measured with a microplate reader (Bio-Rad Laboratories, Hercules, CA).

Immunohistochemical staining was performed as described [35] using anti-TNF monoclonal antibody and anti-CD31 polyclonal antibody. For immunofluorescence, tumor sections were incubated with diluted anti-TNF monoclonal antibodies and anti-CD31 polyclonal antibodies at 4°C overnight, and then treated with rhodamine-conjugated goat anti-mouse IgG and fluorescein isothiocyanate-conjugated goat anti-rabbit IgG at 1:200 for 1 h at room temperature. Sections were analyzed by fluorescence microscopy.

### Evan's blue assay

Twenty days after subcutaneous injection of SGC7901 cells, nude mice were intravenously treated with GX1-rmhTNFα, with rmhTNFα and NS as controls. Two hours later, the mice were intravenously injected with 0.1 ml Evan's blue (Sigma, St. Louis, MO; 12.5 mg/ml). After 5 min, the animals were sacrificed and the tumors were excised. Each tumor was weighed, homogenized, resuspended in cold PBS containing 1% Triton X-100 (1 ml/g), and incubated for 1 h on ice. The suspension was centrifuged (14,000 × g, 4°C, 15 min), and the supernatant mixed with trichloroacetic acid (10%, v/v). The product was centrifuged again (14,000 × g, 4°C, 15 min) and the absorbance at 405 nm of the supernatant was measured using a spectrophotometer.

### *In vivo *tumor formation assay

Seven days after injection of SGC7901 cells as described above, nude mice were randomly divided into groups of seven mice and treated with the indicated reagents on alternate days. Tumor development was observed by sequential caliper measurements of length (L) and width (W). Tumor volume was calculated by the formula L × W^2^/2. After 20 days, the mice were killed and the tumors were removed and weighed. All studies were performed according to internationally recognized guidelines for animal care.

### Statistical analysis

Each experiment was repeated at least three times. Numerical data are presented as mean ± standard deviation (SD). The difference between means was analyzed by ANOVA. All statistical analyses were performed using SPSS11.0 software (Chicago, IL). Differences were considered statistically significant when *p *< 0.05.

## Authors' contributions

BC, SSC designed, carried out the experiments, analyzed data and drafted the manuscript. XLH labeled GX1 with ^99^Tc^m^O_4_^-^, performed the ECT imaging and commented on the manuscript. YW performed Evan's blue assay and commented on the manuscript. WQD performed FCM assay and corrected the final manuscript. LW performed ELISA and commented on the manuscript. XW, JL, YQZ, DMF supervised the project and commented on the manuscript. KCW obtained funding, supervised the project and corrected final manuscript. All authors read and approved the final manuscript.
